# Community-acquired Methicillin-Resistant *Staphylococcus aureus* ST398 Infection, Italy

**DOI:** 10.3201/eid1505.081417

**Published:** 2009-05

**Authors:** Angelo Pan, Antonio Battisti, Alessia Zoncada, Francesco Bernieri, Massimo Boldini, Alessia Franco, Maurilio Giorgi, Manuela Iurescia, Silvia Lorenzotti, Mario Martinotti, Monica Monaci, Annalisa Pantosti

**Affiliations:** Istituti Ospitalieri di Cremona, Cremona, Italy (A. Pan, A. Zoncada, F. Bernieri, S. Lorenzotti, M. Martinotti); Istituto Zooprofilattico Sperimentale delle Regioni Lazio e Toscana, Rome, Italy (A. Battisti, A. Franco, M. Iurescia); Istituto Zooprofilattico Sperimentale delle Regioni Lombardia ed Emilia-Romagna, Cremona (M. Boldini); Azienda Sanitaria Locale di Cremona, Cremona (M. Giorgi); Istituto Superiore di Sanità, Rome (M. Monaci, A. Pantosti)

**Keywords:** Antimicrobial resistance, staphylococci, Staphylococcus aureus, community acquired, MRSA, ST398, pig strain, bloodstream infection, letter

**To the Editor:** Community-acquired methicillin-resistant *Staphylococcus aureus* (CA-MRSA) has been identified in livestock animals (particularly pigs), veterinarians, and animal farm workers (*1*,*2*). CA-MRSA strains from pigs have been classified most frequently within the multilocus sequence type (ST) 398 (*1*) and have been rarely identified as a cause of invasive infection in humans (*1*,*3*,*4*). We report a case of invasive infection in a pig-farm worker in Cremona, Italy, an intensive animal farming area; the infection was caused by MRSA of swine origin, ST398.

The case-patient was a 58-year-old man admitted to a surgical department in Cremona, Italy, on July 30, 2007, because of a 1-week history of fever and intense pain in his right buttock. He worked on a pig farm, was obese, consumed high volumes of wine (1.5 L/day), was taking medication for hypertension, and had not had recent (<5 years) contact with the healthcare system. At the time of hospital admission, he was moderately ill, oriented, and cooperative. His right buttock was extremely painful. He reported neither recent trauma nor anything that would explain infection. Laboratory examination showed increased C-reactive protein (298 mg/L) and leukocytosis (28,000 cells/mm^3^) with neutrophilia (80%). Empiric treatment with intravenous ampicillin-sulbactam was started.

Based on clinical and magnetic resonance imaging data, the diagnosis was cellulitis, pyomyositis, and pelvic multiloculated abscess of the buttock. A needle aspiration of the abscess, guided by computed tomography, was performed. Because of persistent fever (38.5°C), oral ciprofloxacin was added to the patient’s treatment regimen on day 3. Blood and abscess cultures yielded MRSA that was sensitive to glycopeptides, rifampin, linezolid, gentamicin, and mupirocin and resistant to co-trimoxazole, macrolides, clindamycin, and fluoroquinolones. After treatment was switched to vancomycin plus rifampin, the patient’s general condition improved; he was discharged from the hospital after 24 days.

An epidemiologic investigation of the patient’s family and 3 fellow workers and their families was performed; nasal and inguinal swabs were obtained from these 11 persons. Two fellow workers were colonized with *S. aureus*, 1 with methicillin-sensitive *S. aureus* (MSSA) and the other with MRSA. The pig farm, a farrow-to-finish production farm with 3,500 pigs, was screened for MRSA according to guidelines of the European Food Safety Authority (*5*). Dust swabs were taken from 5 areas of the farm; 7 MRSA isolates were detected.

*S. aureus* species identification was confirmed by PCR (*6*). Staphylococcal chromosomal cassette *mec* type (SCC*mec*) was identified by multiplex PCR testing (*7*,*8*). Panton-Valentine leukocidin (PVL) gene detection and *spa* and ST typing were performed as previously described (*9*).

The isolate from the patient belonged to *spa* type t899, was ST398, carried an SCC*mec* type IVa cassette, and was PVL negative. The isolate from the MRSA-colonized worker was a t108 strain carrying SCC*mec* type V. The isolate from the MSSA-colonized worker was identified as t899. The dust swabs yielded 7 isolates: 2 belonged to t899 and carryied SCC*mec* IVa; 5 belonged to t108 and carryied *SCCmec* V. The isolates obtained from the patient, farrowing area 7, and gestation area 1 were indistinguishable (i.e., same *spa* type, SCC*mec t*ype, and ST profile; Table), thus confirming the animal origin of transmission.

**Table:** Main characteristics of *Staphylococcus aureus* isolates identified from persons and pig-farm environment, Cremona, Italy, 2007*

Origin of isolate	Sample type	*nuc/mec*	PVL	*spa* type	*mec* type
Patient	Blood	+/+	–	t899	IVa
Pig worker 1	Nasal swab	+/+	–	t108	V
Pig worker 2	Nasal swab	+/–	–	t899	NA
Farrowing area 5	Dust swab	+/+	–	t108	V
Farrowing area 5	Dust swab	+/+	–	t108	V
Farrowing area 6	Dust swab	+/+	–	t108	V
Farrowing area 7	Dust swab	+/+	–	t108	V
Farrowing area 7	Dust swab	+/+	–	t899	IVa
Farrowing area 8	Dust swab	+/+	–	t108	V
Gestation area 1	Dust swab	+/+	–	t899	IVa

*PVL, Panton-Valentine leukocidin; NA, not applicable.

This case highlights other considerations. First, although the isolate, as expected, was PVL negative, its aggressiveness resembled that of PVL-positive strains. Second, all *S. aureus* isolates identified, MRSA and MSSA, belonged to t899 or t108, within the ST398 group, in agreement with the observation of van Dujkeren et al. (*9*) that ST398 MSSA, a possibly virulent strain (*10*), may acquire different SCC*mec* cassettes relatively easily. Third, ST398 carriage was high (75%) among workers; 2 of 4 were carriers of MRSA ST398 and 1 was a carrier of MSSA ST398. This strain may be a hazard to the health of pig farmers and a possible cause of zoonotic infection. When treating pig farmers for possible staphylococcal infection, healthcare workers should consider using antimicrobial drugs effective against MRSA and should consider the aggressive resistance pattern observed in this case, which was more similar to hospital-acquired strains than to classic CA-MRSA.

The identification of a case of ST398 endocarditis (*4*) and of a nosocomial outbreak of ST398 in the Netherlands (*3*) may support the hypothesis that the scarce number of infections reported so far may be due to the still-limited spread of ST398 among critically ill patients; emergence among pigs is thought to be recent. As observed by Wulf and Voss, the pathogenicity, aggressiveness, or potential spread of ST398 among humans remains to be ascertained (*1*).

In conclusion, attention should be given to the emergence of MRSA strains among animals, and continuous surveillance in humans should monitor the extent of disease from MRSA ST398, especially in areas of intensive animal farming. Collaboration between infectious disease specialists, microbiologists, and epidemiologists, on both the human and the veterinary sides, should be strengthened and readied for appropriate action whenever complex, zoonotic, public health issues occur.
